# The relationship between ultra-processed food consumption and internalising symptoms among adolescents from São Paulo city, Southeast Brazil

**DOI:** 10.1017/S1368980021004195

**Published:** 2022-09

**Authors:** Alexandre Faisal-Cury, Maria Alvim Leite, Maria Mercedes Loureiro Escuder, Renata Bertazzi Levy, Maria Fernanda Tourinho Peres

**Affiliations:** 1 Departamento de Medicina Preventiva da, Faculdade de Medicina da, Universidade de São Paulo, Av. Dr. Arnaldo 455, Cerqueira César, São Paulo, SP 01246903, Brazil; 2 Secretaria de Estado da Saúde de São Paulo, Instituto de Saúde, São Paulo, SP, Brazil

**Keywords:** Ultra-processed foods, Internalising symptoms, Depression, Anxiety, Adolescence, Dietary patterns

## Abstract

**Objective:**

To investigate the association between ultra-processed food (UPF) consumption and internalising symptoms (IS) among adolescents.

**Design:**

It is a cross-sectional study. Paper-pencil survey was completed in classroom with information on UPF consumption, IS and selected covariates. IS were assessed with the Internalizing Symptoms sub-scale from the Social Behaviour Questionnaire (IS-SBQ). UPF was evaluated with a FFQ extracted from the Brazilian National School Health Survey. Crude and adjusted association between UPF and IS was investigated with structural equation models.

**Setting:**

São Paulo, SP, Brazil.

**Participants:**

A total of 2680 students, M_age_ = 14·85; (95 % CI 14·81, 14·88).

**Results:**

UPF consumption was associated with higher scores in IS in the crude (*β* = 0·14; *P* < 0·001) and adjusted (*β* = 0·12; *P* < 0·001) models. The higher the consumption of UPF, the higher is the IS score. The following variables were associated with a lower risk of UPF consumption: male sex, public school and having more meals with parents. The change in the magnitude of the standardised score was almost negligible, but the model was significantly improved with the inclusion of covariates.

**Conclusions:**

Our results provide evidence about the positive association between UPF consumption and IS among adolescents. The association, despite its low magnitude, remained significant after adjusting for potential confounders. These results are relevant considering the increase in UPF consumption worldwide and in low- and middle-income countries. Also, our study emphasises the importance of a healthy diet with a reduction in UPF consumption among adolescents.

Ultra-processed foods (UPF) are formulations of ingredients, mostly of exclusive industrial use, that result from a series of industrial processes, which typically contain little or even no intact food^(1)^. Those products are energy-dense and nutritionally unbalanced. Compared to culinary preparations and processed foods, UPF have, on average, a higher content of free sugar, saturated fat and total fat. They also contain synthetic ingredients, such as flavouring, dyes, texturises, as well as a lower density of proteins, fibres and micronutrients^(2,3)^. Consumption of UPF has exponentially increased worldwide. Data from 79 high- and middle-income countries showed that UPF dominate the food supplies of high-income countries, and that their consumption is now rapidly increasing in middle-income countries^(4)^. According to a report that evaluated the sales of UPF worldwide including thirteen Latin American countries between 2000 and 2013, both retail sales and fast-food transactions increased steadily in almost all countries. In Latin America, most ultra-processed products are increasingly sold in convenience stores, supermarkets and hypermarkets^(5)^.

Several studies have observed a positive association between UPF consumption and increased risk of chronic non-communicable diseases^(1,6)^. There is clear evidence of a negative impact of UPF on functional gastrointestinal disorders^(7)^, asthma in adolescents^(8)^, obesity^(9)^, hypertension^(10)^, metabolic disorders in adolescents^(11)^ and cancer^(12)^.

UPF has also been linked to mental health problems and depression^(13–16)^. In general, a diet rich in fruits, vegetables, olive oil and legumes is protective against mental health disorders^(17)^. In contrast, high intake of saturated fat and refined carbohydrates, typical from a diet with a high participation of UPF, increased the risk of mental disorders^(15,18)^. A study reported that higher levels of diet with components that promote inflammation such as red meat, takeaway and refined foods, soft drink and confectionary were associated with increased risk of depression^(19)^. Few biological pathways have been suggested to explain the link between an unhealthy diet and the onset of depression^(20,21)^. Bioactive compounds of UPF activate inflammatory responses through the release of cytokines and the activation of immune system. Elevated levels of inflammatory markers were described in depressed people^(22)^.

The Internalizing and Externalizing Problems framework was originally conceived by Achenbach in 1991 and it is still frequently used in adolescent’s behaviours studies. Internalising symptoms (IS) refer to problems of withdrawal, somatic complaints and anxiety/depression, while externalising symptoms exhibit themselves in delinquent and aggressive behavior. Both concepts are very helpful for research and treatment purposes^(23,24)^.

The relationship between UPF and mental health has been less studied among adolescents. A prospective cohort in Australia found that a Western dietary pattern characterized by increased intake of takeaway foods, confectionary and red meat was associated with IS, at the age of 14 years^(25)^. In contrast, a prospective Canadian study with 3757 5th grade students (aged 10–11 years) did not find an association between diet quality and internalising disorders. Nevertheless, lower rates of internalising disorders were observed among children with greater variety in their diet^(26)^. A systematic review assessed the association between dietary intake of children and young people and depression. They found a relationship between unhealthy diet and consumption of low-quality diet with depression or poor mental health even though the effect sizes were small^(27)^. Many studies have been carried out in developed countries. However, there is a need for studies in low- and middle-income countries where there are specific cultural and socio-demographic characteristics related to UPF consumption. Moreover, the present study, differently from other publications, emphasised the importance of food processing in a diet pattern more than isolated unhealthy foods or nutrients.

Depression may have a short- and long-term negative impact on several domains of adolescent’s health and social life including academic performance, substance abuse in adult life and risk of suicide^(28)^. Rates of depression increase substantially between 13 and 18 years of age, and the estimated cumulative incidence in adolescents approximates the adult lifetime prevalence rate^(29)^.

The objective of the present study is to investigate the relationship between UPF consumption and IS among adolescents. We hypothesised that UPF consumption is associated with an increased risk of IS.

## Methods

### Study design and sample

For this paper, we used data from the *São Paulo Project on the social development of children and adolescents* (SP-PROSO). SP-PROSO is a cross-sectional study carried on with a representative sample of the 9th grade students in public (municipal and state) and private schools in the city of São Paulo, Brazil, in 2017. São Paulo is the highest city in Brazil and the second highest in Latin America, with an estimated population of 11,966,088 inhabitants in 2017, 6 % (*n* 719 927) between 10 and 14 years of age, and 7 % (*n* 821 019) between 15 and 19 years of age.

The sample size in São Paulo was determined as 2849 students to allow prevalence estimates as lower as 15 % (calculated based on estimates for events of violence with a minimum prevalence of 15 %), with a precision of 0·06 and design effect (deff) = 1·7. To reach this number, we used a stratified sampling procedure, with strata defined according to school administrative domains (public municipal, public state and private), and having classes as primary sampling units. A total of 156 classes, one class per school, were randomly selected, and 119 schools agreed to participate.

Any adolescent present at classroom at data collection day whose parents did not proscribe their child’s participation and who did not present any serious impairments that avoided understanding of the questions or the possibility of answering it anonymously were considered eligible. Of the 2816 students who were present in the classroom, 113 did not participate because they did not meet at least one of the eligibility criteria, which resulted in a sample of 2702 adolescents. For the analysis, twenty-two questionnaires were excluded because more than 20 % of the questions were not answered. Final sample for the present analysis is 2680, 94·1 % of the estimated sample. Data collection was performed on a pre-determined day during classroom time, without the presence of teachers or any other school staff. Students answered the paper-pencil survey anonymously with the support of trained researchers.

Once SP-PROSO was designed to allow for comparative analysis with Z-PROSO, in Zurich, and M-PROSO, in Montevideo, the same instrument and scales were used and translated into Portuguese from English, Germany and Spanish following recommendations for culturally sensitive translations. Additional information about translation can be found at Nivette *et al.* (2020)^(30)^.

### Variables

#### Main outcome

To measure IS, we used the Internalizing Symptoms sub-scale from the Social Behaviour Questionnaire (IS-SBQ)^(31)^. SBQ and its subscales were validated previously in Switzerland and showed to have good reliability, criterion and factorial validity, and invariance during child development^(32)^. The IS-SBQ is expected to have a unidimensional structure. It is composed by eight questions about IS in the month previous to the survey, with answers varying from 1-never to 5-very often in a five-point Likert scale. Questions ask about how often in the previous month the adolescent: (1) was bored; (2) cried; (3) was scared, fearful or anxious; (4) was unhappy, miserable, or distressed; (5) felt alone; (6) could not fall asleep; (7) was sad without knowing why; and (8) was worried.

#### Main exposure

The main exposure is an UPF frequency of consumption score. From a FFQ, we selected five answers in which adolescents were asked how often they ate each kind of ultra-processed product in a regular week: (1) sausages; (2) crackers; (3) packet snacks; (4) treats (sweets, candies and chocolates) and (5) sugary drinks. These products are markers of unhealthy eating. The possible answers ranged from never to 7 d. A score was calculated by the sum of answers, varying from 0 to 35 points. This FFQ is validated^(33)^ and was extracted from the Brazilian National School Health Survey^(34)^. There were foods considered healthy diet markers (fruits, vegetables and beans) and unhealthy diet markers (UPF).

#### Covariates

The following covariates were included for adjusting purposes: sex (male/female) age (in years), skin colour (white/black-brown/yellow (Asian descendants)-indigenous mother schooling (up to complete elementary school, incomplete or complete high school and incomplete or complete higher education) and school administrative dependency (public/private). Physical activity (PA) practice and the habit of having meals with parents (HMP) were also included as adjusting variables, considering they can be both associated with UPF consumption and IS^(35,36)^. PA was measured considering the weekly frequency and duration of active transportation (walking or cycling) to and from school, leisure PA, and participation in physical education classes during the past week, questions extracted from the Brazilian National School Health Survey^(34)^. This questionnaire was previously validated and showed satisfactory relative validity^(37)^. Total PA was calculated adding the min/week of each domain and adolescents we considered physically active if total PA exceeded 299 min/week. HMP was measured by the following question: ‘Do you usually have lunch or dinner with your mother, father or guardian?’ and the adolescents could answer ‘no’, ‘rarely’, ‘1 or 2 d weekly’, ‘3 or 4 d weekly’, ‘5 or 6 d weekly’ or ‘every day’. The answers were dichotomised into ‘4 or less days a week’ and ‘5 or more days a week’. This question was also extracted from the Brazilian National School Health Survey^(34)^.

### Statistical analysis

To evaluate factorial structure, dimensionality and internal consistency of IS-SBQ in Brazilian adolescents, we conducted exploratory factor analysis (EFA), followed confirmatory factor analysis (CFA). To check for adequacy of our data to EFA, the Kaiser–Meyer–Olkin (KMO) index was calculated. KMO estimates the proportion of the set of items variance that could be explained by a latent factor. A KMO ≥ 0·8 was considered meritorious and KMO ≥ 0·9 as excellent. The Bartlett’s test for sphericity tests the null hypothesis of a zero correlation in the sample matrix. A *P*-value ≤ 0·05 is indicative of adequacy of the correlation matrix to conduct EFA.

Once we expect a unidimensional solution, EFA model was run using the principal component factor method^(38)^. The number of factors extracted was determined based on the Kaiser–Guttman rule and the scree test. Kaiser criterion establishes eigenvalue ≥ 1·0 as indicating a non-trivial factor, and the scree test is based on the visual scrutiny of the curve formed by the plotted eigenvalues. The number of factors is determined by the point of greatest change in the slope of the curve^(38)^. Additionally, in this criteria, interpretability and theoretical consistence were also considered. No rotation method was applied based on the resulted simple structure.

A CFA using the structural equations model builder in Stata was run after EFA. For CFA, each of the eight items of IS-SBQ were included in the measurement model as exogenous observable variables, and IS was defined as endogenous latent variable. Maximum likelihood with missing values estimation method (*mlmv*) was used. Modification index was considered to improve model specification with the inclusion of covariance between error terms of exogenous variables. Model fit was evaluated based on Root Mean Square Error of Approximation (RMSEA) (≤ 0·05) and Confirmatory Fit Index (CFI) (≥ 0·95). To measure the scale reliability based on CFA ( 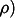 , we refit the unstandardised model fixing the variance of IS. Factorial loadings ( 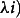 , error variances ( 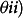 and error terms covariances ( 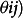 were applied to the following equation:

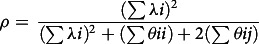




A descriptive analysis was conducted based on the calculation of proportion and 95 % CI for categorical variables, and means and 95 % CI for continuous or discrete variables.

A maximum likelihood missing value (*mlmv)* structural equations model was then run to investigate the association between the observed exogenous variable UPF and the latent endogenous variable IS. *Mlmv* uses full information maximum likelihood techniques to account for missing values^(39)^. Crude and adjusted regression coefficients were calculated. Adjusting observed exogenous variables were sex, age, skin colour, mother’s education, type of school, PA and HMP. Once our sample is complex, sampling weight was used to run the descriptive analysis and the structural equations model. All the analyses were run using Stata 15.1.

## Results

Characteristics of the sample are presented in Table 1, together with the mean score of UPF consumption and IS. UPF consumption is higher among females and students from public schools, and lower among those whose mothers have a higher schooling level. IS mean score is higher among females.

**Table 1:** Sample characteristics (*n* 2680). SP-PROSO, São Paulo, Brazil, 2017

	Total		Ultra-processed food frequency of consumption score		Internalising symptoms scale score	
	Mean	95 % CI	min = 0; max = 35		min = 1; max = 5	
Age	14·85	14·81, 14·88				

	%	95 % CI	Mean	95 % CI	Mean	95 % CI
Sex						
Male	52·60	50·90, 54·29	18·93	18·31, 19·56	2·27	2·23, 2·32
Female	47·4	45·71, 49·10	20·36	19·7, 21·02	2·95	2·89, 3·02
Mother schooling						
Up to complete elementary school	24·78	22·71, 26·98	20·84	20·03, 21·66	2·55	2·46, 2·64
Incomplete or complete high school	40·19	37·52, 42·91	20·64	20·04, 21·23	2·60	2·55, 2·65
Incomplete or complete higher education	35·03	32·06, 38·12	17·73	16·99, 18·47	2·63	2·57, 2·69
Skin colour						
White	44·22	41·68, 46·80	18·13	17·45, 18·81	2·62	2·56, 2·68
Black or brown	49·07	46·26, 51·89	21·06	20·48, 21·64	2·56	2·5, 2·61
Yellow (Asian descendants) or indigenous	6·70	5·51, 8·14	19·84	17·95, 21·72	2·74	2·58, 2·9
School administration						
Private	30·25	28·4, 32·17	16·46	15·62, 17·30	2·66	2·6, 2·71
Public	69·75	67·83, 71·6	21·1	20·6, 21·59	2·57	2·52, 2·62
Physical activity						
< 300 min/week	75·52	73·13, 77·76	19·48	18·97, 19·99	2·65	2·6, 2·7
≥ 300 min/week	24·48	22·24, 26·87	19·89	18·93, 20·84	2·5	2·42, 2·58
Having meals with parents’ frequency						
4 or less d/week	33·94	31·95, 35·98	19·37	18·65, 20·08	2·75	2·67, 2·82
5 or more d/week	66·06	64·02, 68·05	19·79	19·28, 20·3	2·52	2·48, 2·57

Based on KMO (KMO = 0·90) and Bartlett’s test of sphericity test (*P* < 0·001), our data are adequate to conduct EFA, suggesting the existence of a latent factor for IS (Table 2). The first factor explains around 50 % of the shared variance among items. Once one single factor was extracted, no rotation was applied. Cronbach’s *α* (0·84) points to a high internal consistency. As can be seen in Table 2, all items contribute to the highest consistency achieved with the full set of eight items, with smallest values reached as items are excluded. Item-test and Item-rest correlation show the non-negligible correlations.

**Table 2:** Internal consistency and factorial structure of the internalising symptoms SBQ subscale, São Paulo, Brazil, 2017

	Internal consistency			Exploratory factor analysis	
	Item-test correlation	Item-rest correlation	*α*	Factorial loadings	Uniqueness
				Factor 1	
I was bored	0·5477	0·4243	0·8384	0·5376	0·7110
I cried	0·7244	0·6184	0·8159	0·7380	0·4553
I was scared, fearful or anxious	0·7328	0·6279	0·8146	0·7448	0·4453
I was unhappy, miserable or distressed	0·6255	0·4835	0·8336	0·6051	0·6339
I felt alone	0·7777	0·6825	0·8069	0·7827	0·3874
I couldn’t fall asleep	0·6365	0·5072	0·8298	0·6290	0·6043
I was sad without knowing why	0·7834	0·6839	0·8062	0·7900	0·3759
I was worried	0·6550	0·5369	0·8260	0·6669	0·5552
Cronbach’s *α*			0·8407		
Eigenvalue				3·83	
Proportion				0·48	

SBQ, Social Behaviour Questionnaire.

**Fig. 1:**
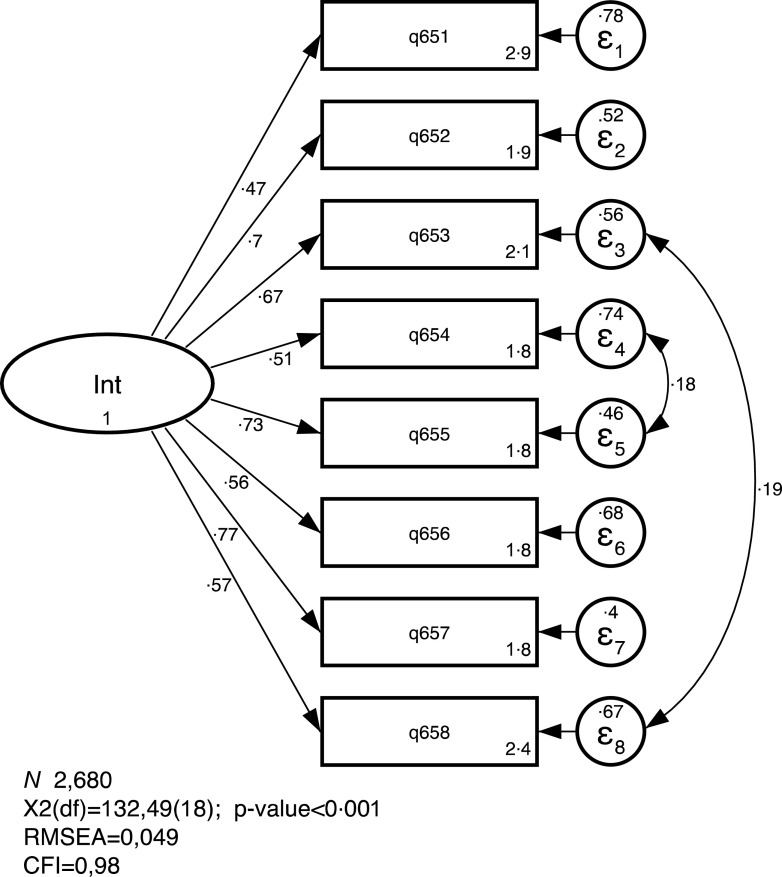
Confirmatory factor analysis for the unidimensional solution of the IS-SBQ. São Paulo, Brazil, 2017 (standardized solution). IS-SBQ, Internalizing Symptoms sub-scale from the Social Behaviour Questionnaire; RMSEA, Root Mean Square Error of Approximation; CFI, Confirmatory Fit Index

The CFA for the unidimensional standardised solution is presented in Fig. 1 and corresponding table can be seen in the Supplemental File. All observed exogenous variables loads significantly with the latent endogenous variable IS. Standardised factor loadings range from 0·47 to 0·77. Covariance between two sets of indicators were included following modification indices, and the final model presents a good fit (*χ* ^2^ (df) = 132·49 (18), *P* < 0·001; RMSEA = 0·049 and CFI = 0·98), confirming the unidimensional solution. The resulting scale reliability is 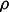 = 0 82.

**Fig. 2:**
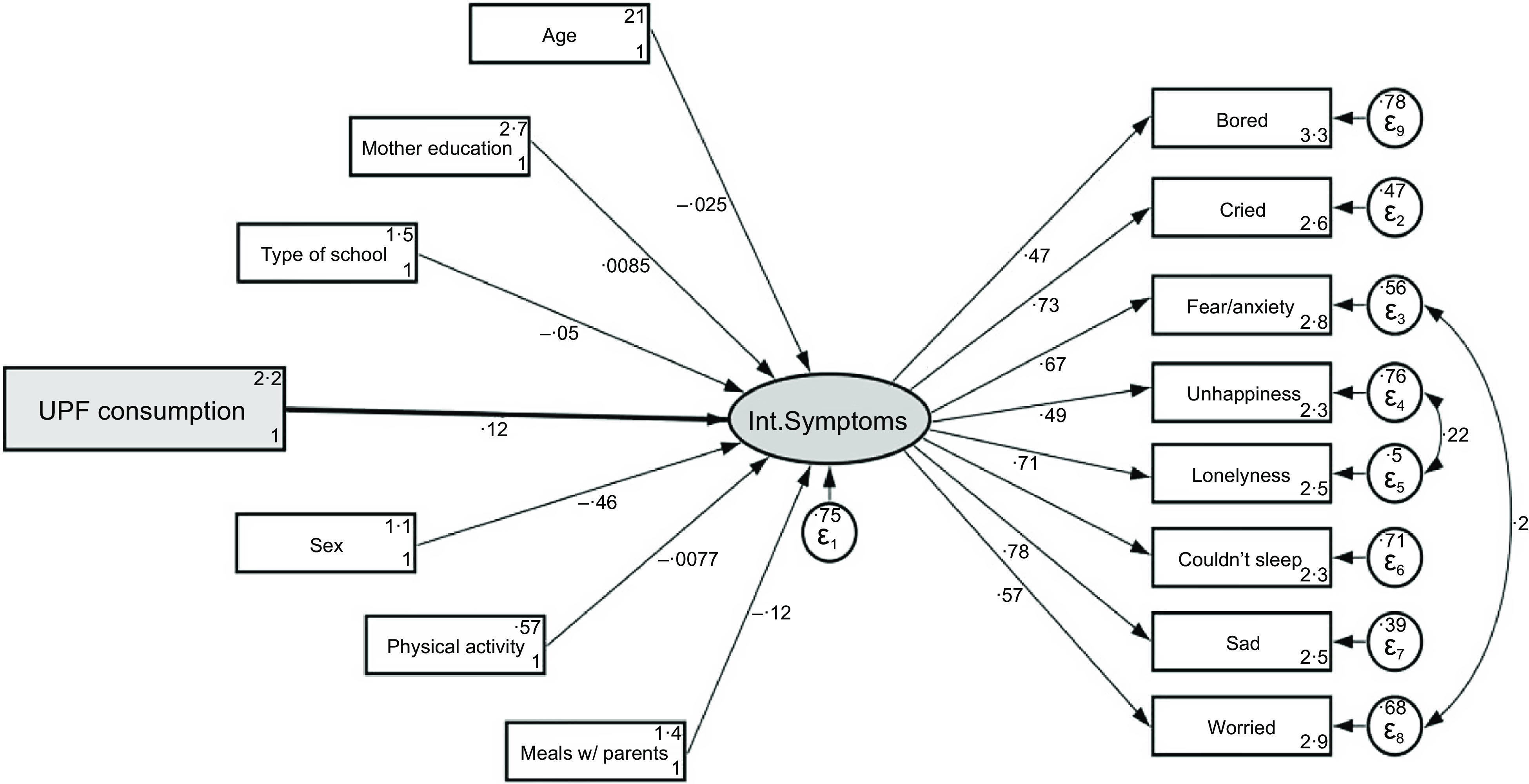
Structural equation models for the adjusted association between ultra-processed food (UPF) consumption and internalising symptoms behaviors among adolescents in São Paulo (*n* 2680), Brazil, 2017

The crude and adjusted associations between UPF consumption and IS can be seen in Fig. 2 and corresponding Table 3. UPF consumption is associated with higher scores in IS in the crude and adjusted models. The higher the consumption of UPF, the higher is the IS score. The change in the magnitude of the standardised score was almost negligible, from 0·14 to 0·12, but the model was significantly improved with the inclusion of covariates, as the coefficient of determination increased from 0·019 to 0·25.

**Table 3:** Structural equation models for the crude and adjusted association between ultra-processed food (UPF) consumption and internalising symptoms behaviours among adolescents in São Paulo (*n* 2680), Brazil, 2017

Coefficients	Crude		Adjusted	
	*β* (stdz)	*β*	*β* (stdz)	*β*
UPF consumption	0·14*	0·01*	0·12*	0·01*
Age	–	–	−0·03	−0·03
Sex (ref = girls)	–	–	−0·46*	−0·88*
Type of school (ref = private)	–	–	−0·05†	−0·11†
Mother schooling (ref = up to complete elementary school)	–	–	0·1	0·01
Physical activity (ref = < 60 min/week)	–	–	−0·007	−0·02
Meals with parentes (ref = 4 or less days a week)	–	–	−0·12*	−0·25*
Coefficient of determination	0·019		0·25	

* *P* < 0·001.

† *P* < 0·05.

## Discussion

Our main findings were the association between female sex, private school, having less meals with parents and higher IS score with a higher risk of UPF consumption. More importantly, the size of the regression coefficient linking the dietary pattern with UPF consumption and IS was small, and the change after controlling for confounding variables was negligible. This result is in agreement with our main hypothesis and has been also reported in other studies performed in high-income countries. This association may be a cause of concern as there is an increase in UPF consumption in several countries and groups, including adolescents^(40,41)^. In Brazil, as other countries, adolescents have the largest share of UPF, in relation to the total energetic intake (26·7 %) (adults have 19·5 % and elderly 15·1 %)^(42)^.

The association between unhealthy dietary patterns, as is the case for consumption of UPF, and depression in adults has been confirmed by many authors. Two large cohort studies in Spain (*n* 14·907, mean age of participants = 36·7 years)^(14)^ and France (*n* 26·730, mean age of participants = 47·2)^(13)^ reached the same conclusion: UPF consumption was associated with the risk of incident depression. A meta-analysis including twenty-one studies conducted in ten countries reported that a high *v*. low ‘Western-type diet’ (rich in red meat, processed meat, sweets, high-fat dairy products, butter, potatoes and high-fat gravy) was associated with an 18 % increased risk of depression^(22)^. Despite this solid evidence, Shivappa *et al.* (2018)^(16)^ questioned if unhealthy diet characterised by a high inflammatory index such as UPF would be a better predictor of depression and depressive symptoms in older than younger people. This assumption relies on the fact that older people have had greater cumulative exposure to an inflammatory diet than younger subjects during their lives. Despite the pathophysiological plausibility, this hypothesis is not supported by the results of studies with adolescents.

The Western Australian Pregnancy Cohort (Raine) Study evaluated the relationship between dietary patterns and mental health in early adolescence. Two dietary patterns (Western and Healthy) were identified using factor analysis and food group intakes estimated by a 212-item FFQ. They employed the Child Behaviour Checklist (CBCL) to assess mental health status among 1324 adolescents (mean age of 14 years). Internalising and externalising CBCL scores were significantly associated with the Western dietary pattern, with increased intakes of takeaway foods, confectionary and red meat being associated with higher scores in CBCL. In contrast, improved behavioural scores were significantly associated with higher intakes of leafy green vegetables and fresh fruit (components of a healthy pattern)^(25)^. Of note, in the adjusted model, the Western dietary pattern was more strongly associated with externalising symptoms than for IS. Khalid *et al.* (2016)^(27)^ performed a systematic review evaluating the relationship between diet and mental health in youth. They included twenty articles being seventeen cross-sectional and three prospective cohort studies with follow-up periods ranging from 2 to 4 years. Overall, they found an association between healthy dietary patterns or consumption of a high-quality diet and lower levels of depression or better mental health. Similarly, there was a relationship between unhealthy diet and consumption of low-quality diet and depression or poor mental health. However, where significant relationships were reported, effect sizes were small. Moreover, most studies used a cross-sectional design, attempted to control the impact of confounders and included multiple measures of diet quality and multiple testing^(27)^.

One possible mechanism explaining the relationship between UPF and mental health lies on the role of inflammation. Bioactive compounds of diet may exhibit different inflammatory properties^(20)^. Poor diet as well many other factors (stress, smoking, physical inactivity, etc.) may increase the risk of depression through an activation of systemic inflammation which is the result of the release of proinflammatory cytokines from immune cells and chronic activation of the innate immune system^(21)^. Depressed people have elevated levels of various inflammatory markers including c-reactive protein, IL-6 and TNF^(43)^. Several mechanisms explain how systemic inflammation affects the brain and mood regulation^(21)^. Nevertheless, cross-sectional studies cannot exclude an alternative explanation for the association between UPF and IS. It is also possible that IS drive the eating behaviour. Studies on rodents explained how chronic stress (and high concentration of glucocorticoids) leads to a direct excitatory effect on brain that motivates ingestion of ‘comfort food’^(44)^. The effects chronic stress (and glucocorticoids) seen in rats are possible applied to humans. Studies showed that individuals with clinical or subclinical psychiatric disorders often overeat as a reaction to distress^(45)^. Additionally, there are evidence that high-fat and carbohydrate foods may help people to feel and function better^(46)^.

Our data support the importance of making efforts to improve dietary patterns among adolescents, given its important public health association on mental health beside other non-communicable chronic diseases. These healthy dietary patterns are based on the degree and purpose of food processing and recognises the value of unprocessed and minimally processed foods^(1)^. In particular, dietary intake is a modifiable associated factor for depression and anxiety. Globally, depression is the fourth leading cause of illness and disability among adolescents aged 15–19 years and fifteenth for those aged 10–14 years. Anxiety is the ninth leading cause for adolescents aged 15–19 years and sixth for those aged 10–14 years. Emotional disorders can profoundly affect areas like schoolwork and school attendance. At its worse, depression can lead to suicide^(47)^. In Brazil, a large cross-sectional, national, school-based study conducted in 2013–2014 with 74 589 adolescents found a 30 % prevalence of common mental disorders^(48)^. Common mental disorders are mainly characterized by the presence of symptoms of depression and anxiety, and various non-specific and somatic complaints.

Our study has some strengths. First of all, to our knowledge, this is the first Brazilian study to examine the relationship between consumption of UPF and mental health in a large sample of adolescent population. Importantly, this association was controlled for a variety of potential confounders, including PA, doing meal with parents and socio-demographic factors. Regarding our outcome, we used a valid and reliable measure of IS. Moreover, we performed an internal validation of a latent variable. The instrument used for the evaluation of food consumption frequency was previously validated assuring the quality of dietary data assessment^(33)^. External validity is another positive aspect of our study, since it showed a high response rate in a representative sample of 9th grade students from public and private schools. According to the Continuous National Household Sample Survey (PNAD), 97·5 % of children and adolescents, with compatible age, would be enrolled in elementary school in the city of São Paulo in 2017^(49)^. In addition, the self-completion and comprehension of the questionnaire are highly expected at this group of adolescents. Other relevant aspect of the present study is the focus on food processing – and not in isolated nutrients – as defined by NOVA classification, which classifies foods according to the degree and purpose of industrial processing, and brings the UPF concept^(1)^. We also believe that in terms of a public health policy is probably better to inform the population to decrease the ingestion of UPF in general than to reduce specific nutrients and foods.

Nevertheless, our study has some limitations. Firstly, the cross-sectional design does not allow to establish causal relationships. We cannot exclude a strong possibility of reversal causality nor the possible cyclic nature of the association between UPF and IS. It is possible that low mood leads to increased consumption of UPF or that both reinforce each other in a cyclic process of reinforcement. Secondly, our assessments were based on self-report instruments. Although valid, they are subject to some degree of misclassification and subjectivity. Moreover, it is important to mention that we used a part of the FFQ to assess UPF consumption, not evaluating the whole diet of the adolescents. These UPF, however, are markers of unhealthy eating and are between main industrialised products consumed by Brazilian adolescents^(42)^. Concerning validity and reliability of the IS-SBQ scale, even though it was not previously validated in Brazil and ideally, validity and reliability should had been evaluated in a distinct sample, we conduct EFA and CFA, confirming its unidimensional solution and excellent reliability, making us confident about its adequacy to our adolescent population as previously described for Switzerland adolescents^(32)^. In this sense, one further limitation is the use of a questionnaire for the evaluation of IS among adolescents. Ideally, we should use a diagnostic criterion for depression classification. Nevertheless, the large sample size limits this option. Third, once our survey was school-based and the target population were adolescents present at school on data collection day, only adolescents enrolled at schools and present at classes were included. Also, some schools refused to participate. These could result in selection bias. Unfortunately, we do not have information about adolescents that are out of school and about the schools that refuse to participate, what could give us additional information to clearly evaluate the impact of a potential bias. It should be stated however that the number of unrolled adolescents is small. Once truant adolescents are more prone to risky habits and lower health, it is possible to suppose that those who are out of school have both a higher UPF and IS. If so, even though the magnitude of prevalence could be underestimated, no change would be expected in the measure of association. Fourth, considering that our study was performed with young students from Sao Paulo city, our results cannot be generalised to other groups. Finally, there is a risk of additional non-adjusted confounding, such as family history of depressive disorders, stressful life events and sleep disorders that are associated with depression^(50)^.

Our findings support a relationship between consumption of UPF and higher risk of IS among adolescents aged 13 to 17 years. Considering possible ethical implications of a randomised trial for the assessment of the negative impact of UPF on adolescents’ mental health, further observational longitudinal studies are recommended.

## Supplementary material

For supplementary material accompanying this paper visit https://doi.org/10.1017/S1368980021004195. click here to view supplementary material
