# Supplementary material for: The relationship between ultra-processed food consumption and internalising symptoms among adolescents from São Paulo city, Southeast Brazil
Source: Public Health Nutr. 2021 Oct 6;25(9):2498–506. doi: 10.1017/S1368980021004195 (PMC9991766; doi:10.1017/S1368980021004195)
Supplement: Supplementary file 1 [file S1368980021004195sup001.docx]

Supplementary table: Confirmatory Factor Analysis for the unidimensional solution of the ISs-SBQ. São Paulo, Brazil, 2017 (Standardized and unstandardized solutions).

|  | Standardized | |  | Unstandardized | |
| --- | --- | --- | --- | --- | --- |
| Loadings | ß | 95%CI |  | B | 95%CI |
| X_1_:I was bored | 0.47^***^ | 0.44;0.50 |  | 1 (fixed) |  |
| X_2_:I cried | 0.70^***^ | 0.67;0.72 |  | 1.72^***^ | 1.57;1.88 |
| X_3_:I was scared, fearful, or anxious | 0.66^***^ | 0.64;0.69 |  | 1.67^***^ | 1.51;1.82 |
| X_4_:I was unhappy, miserable, or distressed | 0.51^***^ | 0.47;0.54 |  | 1.34^***^ | 1.20;1.48 |
| X_5_:I felt alone | 0.73^***^ | 0.71;0.76 |  | 1.91^***^ | 1.75;1.08 |
| X_6_:I couldn't fall asleep | 0.56^***^ | 0.53;0.59 |  | 1.40^***^ | 1.26;1.54 |
| X_7_:I was sad without knowing why | 0.77^***^ | 0.75;0.79 |  | 2.12^***^ | 1.93;2.30 |
| X_8_:I was worried | 0.57^***^ | 0.54;0.60 |  | 1.36^***^ | 1.22;1.49 |
| Variances |  |  |  |  |  |
| e.X_1_ | 0.78 |  |  | 1.01 |  |
| e.X_2_ | 0.52 |  |  | 0.91 |  |
| e.X_3_ | 0.56 |  |  | 1.00 |  |
| e.X_4_ | 0.75 |  |  | 1.51 |  |
| e.X_5_ | 0.46 |  |  | 0.90 |  |
| e.X_6_ | 0.68 |  |  | 1.22 |  |
| e.X_7_ | 0.40 |  |  | 0.87 |  |
| e.X_8_ | 0.67 |  |  | 1.09 |  |
| Internalizing Symptoms | 1 (fixed) |  |  | 0.29 |  |
| Covariance |  |  |  |  |  |
| e.X_1_ with e.X_8_ | 0.19^***^ | 0.15;0.23 |  | 0.20^***^ |  |
| e.X_4_ with e.X_5_ | 0.18^***^ | 0.14;0.23 |  | 0.21^***^ |  |
| Chi2 (df=18) | <0.001 | | | | |
| RMSEA | 0.049 (p-close=0.586) | | | | |
| Comparative Fit Index (CFI) | 0.98 | | | | |
| Tucker-Lewis index (TLI) | 0.97 | | | | |
| Coefficient of determination | 0.84 | | | | |

CI = Confidence Interval

*** p<0.001
